# Screening and brief intervention (SBI): has it hit the tipping point?

**DOI:** 10.1186/1940-0640-7-14

**Published:** 2012-08-28

**Authors:** Richard Saitz

**Affiliations:** 1 Boston University Schools of Medicine and Public Health/Boston Medical Center, Boston, MA, USA

## 

In 1961, Chafetz [1] reported the results of a randomized trial of brief advice by a psychiatrist to patients with alcoholism in the Massachusetts General Hospital (MGH) emergency department (ED); 42% of patients in the advice arm reported to an alcohol clinic versus only 1% in the control group. Fifty years later, in the Liberty Hotel, the same space as the former Charles Street Jail in Boston where there was a “drunk tank” and across from that same MGH ED, over 200 researchers and clinicians gathered to present over 100 abstracts and plenary sessions on screening and brief intervention (SBI). In those 50 years, thousands of patients participated in randomized trials of SBI; the US Institute of Medicine (in 1990) encouraged identification and intervention for unhealthy alcohol use for people across the spectrum from risky use through dependence; the World Health Organization validated assessment tools and showed SBI’s effectiveness in primary care settings; and the US Substance Abuse and Mental Health Services Administration allocated substantial funding for SBI for both alcohol and other drugs across many general health settings.

Research presented at the September 2011 International Network on Brief Interventions for Alcohol and Other Drugs (INEBRIA) conference in Boston was from around the globe, covered alcohol and other drugs, crossed a variety of health settings and practitioners, and showed the sophistication that has been reached in the field. Research discussed when, where, and for whom SBI has or might not have efficacy, how to implement SBI programs, adaptations of SBI, costs and effectiveness, and many other topics. Nonetheless, despite the excitement, breadth, and sophistication, the fact remains that few patients eligible for SBI receive the service, and, as a result, opportunities to improve health and save health care costs are missed. Most people with alcohol and drug use disorders receive no treatment.

At least in some cases, SBI is ready for dissemination. And, with solid evidence available, health reforms promised in the US and elsewhere, and an international discussion taking serious shape regarding the integration of care for medical, mental health, and substance use conditions, SBI may have reached a tipping point for dissemination and implementation as well as for small- and large-scale studies of remaining efficacy and effectiveness questions.

The editors of ASCP invited INEBRIA attendees and presenters to submit their studies for peer review and possible publication. The initial core of this thematic series, “Screening and brief intervention for unhealthy alcohol and other drug use,” is the result of that call for papers, a seed that we expect will grow as the SBI literature continues to become more robust. Papers will cover a range of topics from what the efficacy of SBI really is, to effectiveness in people with mental health and drug use conditions, to adolescent SBI, to health professional attitudes towards SBI, to SBI implementation research and even to state-of-the-art SBI research protocol design, among others. We hope this series begins a long and serious conversation.
